# Draft Genome Report of *Bacillus altitudinis* SORB11, Isolated from the Indian Sector of the Southern Ocean

**DOI:** 10.1128/genomeA.00339-17

**Published:** 2017-06-08

**Authors:** Urmi Halder, Aparna Banerjee, Vasvi Chaudhry, Rajeev K. Varshney, Shrikant Mantri, Rajib Bandopadhyay

**Affiliations:** a UGC-Center of Advanced Study, Department of Botany, The University of Burdwan, Burdwan, India; b CSIR-Institute of Microbial Technology, Chandigarh, India; c Centre of Excellence in Genomics, International Crops Research Institute for the Semi-Arid Tropics (ICRISAT), Patancheru, India; d National Agri-Food Biotechnology Institute (NABI), S.A.S. Nagar, Mohali, India

## Abstract

Here, we present the draft genome sequence of *Bacillus altitudinis* SORB11, which is tolerant to UV radiation. The strain was isolated from the Indian sector of the Southern Ocean at a depth of 3.8 km. The genome sequence information reported here for *B. altitudinis* SORB11 gives the basis of its UV resistance mechanism and provides data for further comparative studies with other bacteria resistant to UV radiation.

## GENOME ANNOUNCEMENT

The species *Bacillus altitudinis* was first reported from an air sample collected at 41 km in the stratosphere (1). Additional reports revealed its presence in other habitats, such as soil (2), silt (3), fish (4), and plant rhizosphere (5). Here, we present the draft genome sequence of *B. altitudinis* SORB11, which was collected from the Indian sector of the Southern Ocean (50°S, 47°E) at a depth of 3.8 km. To date, 57 bacterial isolates have been reported from the Indian sector of the Southern Ocean (6), but so far there has been no report of *B. altitudinis* from the Southern Ocean.

Genomic DNA of *Bacillus altitudinis* SORB11 was isolated using the Zymo research fungal/bacterial DNA mini prep kit (D6005), and the purity of the final DNA concentration was determined using NanoDrop 1000 version 3.1.1 (Thermo Scientific, USA). Prior to library construction, the quality and quantity of the DNA were checked using agarose gel electrophoresis and the Qubit fluorometer. Libraries were constructed from 1 µg of genomic DNA using the Illumina TrueSeq DNA PCR-free HT library preparation kit. DNA fragmentation was performed using Covaris, followed by end repair and adapter ligation. Final libraries were denatured and sequenced on the Illumina MiSeq (Illumina, USA) platform (7). Sequence data consisted of 3,692,443 reads with an *N*_50_ value 54,515 and 19× coverage. The FastQC tool was used for quality checking. Reads were assembled into contigs by CLC Assembly Cell. Genome annotation was performed using the RAST server (8).

The whole genome of SORB11 is 3,692,443 bp in size with a 41.2% mean GC content. Based on a complete 16S rDNA sequence analysis of the genome, SORB11 showed 100% similarity to type strain *B. altitudinis* 41KF2b. Moreover, calculations for average nucleotide identity (http://www.ezbiocloud.net/tools/ani) and DNA–DNA hybridization (http://ggdc.dsmz.de) showed 98.4% and 86% values, respectively, confirming the species status of *B. altitudinis* SORB11. A total of 3,867 coding genes were annotated using the RAST server, with 2 rRNAs and 22 tRNAs.

RAST subsystem information showed a total of 102 genes belonging to DNA metabolism, out of which 55 were associated with DNA repair: 1 uracil-DNA glycosylase; 3 DNA repair, bacterial MutL-MutS system; 3 DNA repair, UvrABC system; 1 DNA repair, bacterial photolyase; 2 DNA repair, bacterial DinG and relative; 2 DNA repair system, including RecA, MutS, and a hypothetical protein; 2 ATP-dependent nuclease; 18 DNA repair, bacterial; 8 DNA repair, bacterial RecFOR pathway; 1 DNA repair, bacterial RecBCD pathway; 2 nonhomologous end-joining in bacteria; 10 DNA repair base excision; and 2 DNA repair, bacterial UvrD and related. Further, a total 103 genes encoding stress response were identified: 13 osmotic stress; 38 oxidative stress; 3 cold shock; 17 heat shock; 8 detoxification; 1 periplasmic stress and 23 other stress response genes. These genes together may confer UV-B resistance in SORB11.

Interestingly, the species *B. altitudinis* has been isolated from extreme habitats, namely, at a height of 41 km in the stratosphere and at a depth of 3.8 km in the Indian sector of the Southern Ocean. Further comparative genome analysis of the diverse *B. altitudinis* strains reported to date will help in delineating the evolutionary branch point of stratospheric microorganisms. Also, genome mining of the genes responsible for the UV resistance of *B. altitudinis* will help in developing a better understanding of stratospheric bacteria and their adaptations in extreme UV-stressed environments.

### Accession number(s).

The whole-genome sequence of *B. altitudinis* SORB11 has been deposited at GenBank under the accession number MEHW00000000.1.
